# SARS-CoV-2 infection risk among 77,587 healthcare workers: a national observational longitudinal cohort study in Wales, United Kingdom, April to November 2020

**DOI:** 10.1177/01410768221107119

**Published:** 2022-07-07

**Authors:** Joe Hollinghurst, Laura North, Tamas Szakmany, Richard Pugh, Gwyneth A Davies, Shanya Sivakumaran, Rebecca Jarvis, Martin Rolles, W Owen Pickrell, Ashley Akbari, Gareth Davies, Rowena Griffiths, Jane Lyons, Fatemeh Torabi, Richard Fry, Mike B Gravenor, Ronan A Lyons

**Affiliations:** 1 Population Data Science and Health Data Research UK, Swansea University Medical School, Swansea University, Swansea, Wales, United Kingdom, SA2 8PP; 2 Critical Care Directorate, Grange University Hospital, Aneurin Bevan University Health Board, Llanyravon, Cwmbran, NP44 2XJ; 3 Department of Anaesthesia, Intensive Care and Pain Medicine, Division of Population Medicine, Cardiff University, Heath Park Campus, Cardiff, CF14 4XN; 4 Department of Anaesthetics, Glan Clwyd Hospital, Betsi Cadwaladr University Health Board; 5 Digital Workforce, NHS Wales Shared Services Partnership; 6 South West Wales Cancer Centre, Singleton Hospital, Swansea SA2 8QA; 7 Swansea University Medical School and Neurology Department, Morriston Hospital, Swansea Bay University Health Board

**Keywords:** COVID-19, SARS-CoV-2, healthcare workers, infection risk, public health

## Abstract

**Objectives:**

To better understand the risk of severe acute respiratory syndrome coronavirus 2 (SARS-CoV-2) infection among healthcare workers, leading to recommendations for the prioritisation of personal protective equipment, testing, training and vaccination.

**Design:**

Observational, longitudinal, national cohort study.

**Setting:**

Our cohort were secondary care (hospital-based) healthcare workers employed by NHS Wales (United Kingdom) organisations from 1 April 2020 to 30 November 2020.

**Participants:**

We included 577,756 monthly observations among 77,587 healthcare workers. Using linked anonymised datasets, participants were grouped into 20 staff roles. Additionally, each role was deemed either patient-facing, non-patient-facing or undetermined. This was linked to individual demographic details and dates of positive SARS-CoV-2 PCR tests.

**Main outcome measures:**

We used univariable and multivariable logistic regression models to determine odds ratios (ORs) for the risk of a positive SARS-CoV-2 PCR test.

**Results:**

Patient-facing healthcare workers were at the highest risk of SARS-CoV-2 infection with an adjusted OR (95% confidence interval [CI]) of 2.28 (95% CI 2.10–2.47). We found that after adjustment, foundation year doctors (OR 1.83 [95% CI 1.47–2.27]), healthcare support workers [OR 1.36 [95% CI 1.20–1.54]) and hospital nurses (OR 1.27 [95% CI 1.12–1.44]) were at the highest risk of infection among all staff groups. Younger healthcare workers and those living in more deprived areas were at a higher risk of infection. We also observed that infection rates varied over time and by organisation.

**Conclusions:**

These findings have important policy implications for the prioritisation of vaccination, testing, training and personal protective equipment provision for patient-facing roles and the higher risk staff groups.

## Introduction

The emergence of severe acute respiratory syndrome coronavirus 2 (SARS-CoV-2), which causes COVID-19, has led to a global health emergency with over 100 million cases and 2 million deaths reported worldwide as of 29 January 2021.^1^ Healthcare workers are at high risk of SARS-CoV-2 infection since their work can require close exposure to infected patients. Globally, healthcare workers account for one in seven COVID-19 cases reported to the World Health Organization.^2^ Universal use of personal protective equipment (PPE) is now viewed as important in reducing transmission, and studies report reductions in rates of COVID-19 among healthcare workers after the introduction of universal masking policies.^3,4^ There is also evidence that in the UK, high availability and consistent use of PPE in areas such as intensive care units prevented viral transmission in these high-risk areas.^5^ However, early in the pandemic, messaging regarding PPE was inconsistent and there were challenges with the imbalance in supply and demand.

It is imperative to understand infections among healthcare workers, since once infected, healthcare workers may continue to work while asymptomatic or pre-symptomatic.^6,7^ Thus, there is the potential for secondary transmission to the vulnerable patients they care for, as well as their colleagues, leading to continued nosocomial spread, a substantial component of the pandemic.^8–10^ Understanding which healthcare workers have the highest risk of contracting infection could help with workforce planning, targeted testing and vaccine prioritisation, as well as risk mitigation in the case of future novel pathogens. Previous studies investigating the risk of SARS-CoV-2 infection among healthcare workers are difficult to interpret due to cross-sectional designs, single-centre inclusion or population selection but have shown significant levels of infection even among healthcare workers not working directly with COVID-19 patients.^11^ There is also evidence of differential risk with different healthcare roles.^6,7,12^ The lack of associated demographic data in some of these studies may also introduce confounding. Population-based studies have assessed the risk of healthcare workers developing severe (hospitalised) COVID-19, but did not examine the risk of becoming infected.^13–15^

In Wales, our national database of healthcare workers employed by the National Health Service (NHS), can be linked anonymously to pathology and demographic data within the Secure Anonymised Information Linkage databank.^16–18^ This enables anonymous up-to-date longitudinal evaluation of the risk of SARS-CoV-2 infection among healthcare workers. We sought to investigate the risk of SARS-CoV-2 infection for patient-facing healthcare workers and to determine which roles in healthcare are associated with the highest risk of acquiring infection.

## Methods

### Study design and setting

We conducted an observational, longitudinal, national cohort study. This included 577,756 monthly observations among 77,587 healthcare workers employed by NHS Wales (UK) organisations during the COVID-19 pandemic. These healthcare workers do not include the majority of primary care (general practice) staff who are employed by hundreds of individual practices, or staff employed via private agencies. We included data from 1 April 2020 to 30 November 2020.

Our cohort was dynamic and healthcare workers were included for each month they were working. We had a maximum of eight observations for each worker, if they were working in each month of the study, and a minimum of one if they were only working in a single month. For each month of study, we observed whether an individual was recorded as having a positive SARS-CoV-2 test. All data were collected routinely and accessed anonymously via the Secure Anonymised Information Linkage Databank.

### Participants

The participants in our study were all healthcare workers employed by NHS Wales (UK). The NHS Electronic Staff Record contains hundreds of specific roles, many with small numbers. For analyses by role, we combined some categories (e.g. different grades of hospital nurses, see Tables S1 and S2 for detailed categorisations) and created groups of healthcare workers for subsequent analyses (Table 1).

**Table 1.** Grouped staff roles for healthcare workers.

• Allied Health Professionals	• Laboratory Staff
• Call Handler	• Manager
• Clerical Worker	• Medical Consultant
• Community Nurse	• Medical Secretary
• Cook	• Medical Student
• Driver	• Middle Grade Doctor
• Foundation Year Doctor	• Midwife
• Healthcare Support Worker	• Paramedic
• Hospital Nurse	• Porter
• Housekeeper	• Student Hospital Nurse

Based on a description of the roles and discussions with clinicians and human resource experts, we further classified the staff roles into patient-facing, non-patient-facing or undetermined. The classifications are detailed in Table S2.

### Data sources/measurement

We used linked longitudinal data from the Secure Anonymised Information Linkage Databank to create our datasets.^16–18^ Specifically, we used the Health Care Workers Database, which is derived from the NHS Electronic Staff Record databases of all secondary care employees submitted to Welsh Government. This is a comprehensive list of all direct secondary care employees of NHS Wales. We used the Health Care Workers Database to indicate who was a healthcare worker, their role, which organisation they worked for and which months they were actively working. The Pathology COVID-19 Daily data were used to record dates of positive SARS-CoV-2 PCR tests. A cleaned and pre-linked version of the Welsh Demographic Service Dataset was used to determine demographic information for each individual.^19^

### Variables

The primary outcome was a positive SARS-CoV-2 polymerase chain reaction (PCR) test. This was observed as a binary yes/no for each month of study using the Pathology COVID-19 Daily (PATD) data. We included the month of observation as a proxy for the change in COVID-19 prevalence over the study period. Month of observation (reference group: April) was included as a categorical variable.

Additional covariates were staff role and whether patient-facing, employing organisation and demographic information. Staff roles and organisation were included as binary (yes/no) dummy variables. This was to ensure that individuals who had multiple organisation affiliations and staff roles were able to be simultaneously included in the analyses. For example, an individual may have been recorded in a single month as both a community nurse and hospital nurse. We found that approximately 4.5% of our cohort had multiple roles recorded. Unfortunately, we were unable to determine the specific time allocation to each role. The grouped staff roles used in the analyses are included in Table 1. The employing organisations were: University Health Boards/Trusts: Betsi Cadwaladr, Cardiff and Vale, Aneurin Bevan, Swansea Bay, Cwm Taf Morgannwg and Hywel Dda; Powys Teaching Health Board, Velindre NHS Trust, Welsh Ambulance Services NHS Trust, NHS Wales Shared Services Partnership, Public Health Wales, NHS Wales Informatics Service, Health Education and Improvement Wales and Single Lead Employer. Each employing organisation was randomly anonymised using a code letter to mask their identity. Patient-facing status was included as a categorical variable (reference group: non-patient-facing). Demographic information included sex (reference group: female), age (continuous) and area-based socioeconomic deprivation quintile (reference group: 1, most deprived). Deprivation quintiles were measured using the Welsh Index of Multiple Deprivation (WIMD) 2019, with quintile 1 being the most deprived and quintile 5 being the least deprived. The WIMD is a weighted score from eight domains assigned to each of the 1909 lower-layer super output areas (LSOAs) in Wales containing an average of 1600 people. Each LSOA has been ranked from most deprived to least deprived according to its WIMD score and then grouped into quintiles.^20^ We used an individual’s home addresses to derive the deprivation quintile. All variables varied with time (per month of observation) to ensure that any changes in roles, organisation or demographics were captured in the dataset.

### Bias

**Figure 1.**
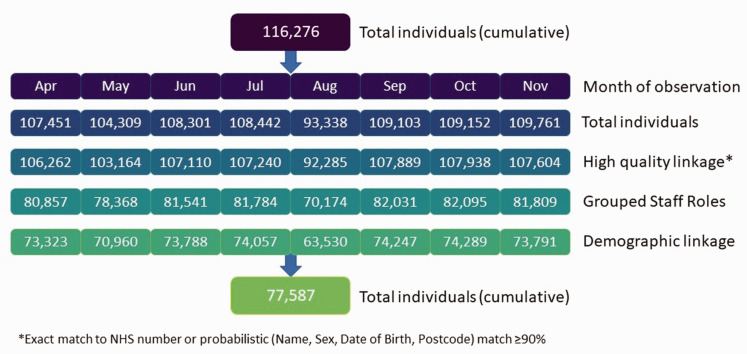
CONSORT diagram for study size and cohort linkage.

We included multiple observations per person to account for changes in role, organisation and exposure time within a healthcare setting. Observations with missing demographic information (sex, WIMD) were excluded; the number of missing observations is recorded in Figure 1. We included all linked individuals to limit selection bias.

### Statistical methods

We used logistic regression models with a logit link to investigate the odds of a positive SARS-CoV-2 PCR test. We included a fixed effect term for each observation month, with up to eight observations per person. As a sensitivity analysis to determine if a random effect was required to account for repeated measures in individuals, we computed two independent multilevel logistic regression models with random intercepts for the month of observation and individual. Differences in the number of positive tests between those with and without demographic information were tested using chi-square tests. Statistical analyses were performed using R version 4.0.0 and R2MLwiN.^21^

## Results

### Participants

We included all healthcare workers with complete demographic information who were identified as having a role contained in Table 1. Only individuals with high-quality linkage were included, consisting of either an exact match or a probabilistic match ≥90%. Further details of the matching procedure have been reported previously.^17^ Chi-square tests indicated no significant difference in the proportions of positive tests between those with and without linked demographic data (Table S3b).

### Descriptive data

**Figure 2.**
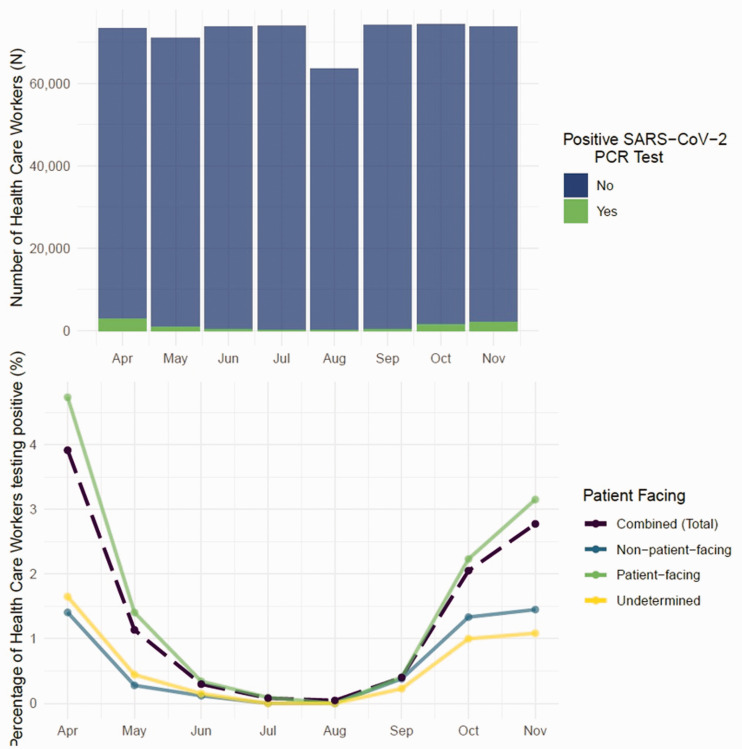
(top) Number of healthcare workers and those testing positive for SARS-CoV-2. (bottom) Percentage of healthcare workers testing positive (combined) with stratifications for patient-facing status (patient-facing, non-patient-facing, undetermined). For illustrative purposes, where exact counts were masked or omitted in July and August a value of 0% was used.

We included 77,587 individuals in our cohort (Figure 1). The number of individuals and positive SARS-CoV-2 PCR tests by staff role per month are recorded in Table 2. Table S4 shows the positive rate of infection by grouped staff role and patient-facing status. This is the number of healthcare workers with a positive test per total number of healthcare workers in each category in each month (e.g. in April, 144 Allied Health Professionals tested positive from a total of 5895, giving a rate of 2.44%). Healthcare workers with patient-facing roles had the highest rate of infection. Foundation year doctors, hospital nurses and healthcare support workers were among those with the highest rates of infection. Figure 2 shows (top) the number of healthcare workers and those with positive SARS-CoV-2 PCR tests per month and (bottom) the percentage of healthcare workers testing positive with stratification for patient-facing status over time.

**Table 2.** Demographic information stratified by observation month and positive SARS-CoV-2 PCR test (yes/no).

Month	Apr	Apr	May	May	Jun	Jun	Jul	Jul	Aug	Aug	Sep	Sep	Oct	Oct	Nov	Nov
SARS-CoV-2 positive PCR test	No	Yes	No	Yes	No	Yes	No	Yes	No	Yes	No	Yes	No	Yes	No	Yes
N	70,476	2847	70,163	797	73,577	211	74,002	55	63,502	28	73,961	286	72,828	1461	71,820	1971
Total tests	4520	2942	4382	846	5041	235	6479	82	4387	32	9873	362	11,366	1909	12,801	2461
**Patient-facing**																
Patient-facing	51,518	2560	51,486	734	54,294	186	54,675	50	46,502	Omitted	54,531	222	53,503	1222	52,635	1713
Non-patient-facing	12,404	177	12,168	34	12,665	15	12,778	<10	11,142	Omitted	12,844	49	12,792	173	12,709	187
Undetermined	6554	110	6509	29	6618	10	6549	<10	5858	Omitted	6586	15	6533	66	6476	71
Grouped staff roles																
Allied Health Professionals	5751	144	5754	49	5930	19	6013	<10	5060	<10	6065	10	6014	60	5930	102
Call Handler	827	32	856	<10	850	<10	852	<10	854	<10	835	<10	849	13	877	<10
Clerical Worker	7436	93	7094	22	7655	<10	7697	<10	6326	<10	7758	31	7692	114	7638	115
Community Nurse	2566	81	2530	16	2635	<10	2648	<10	2087	<10	2627	<10	2589	33	2545	62
Cook	484	18	492	<10	502	<10	490	<10	472	<10	486	<10	502	<10	487	<10
Driver	346	<10	290	<10	354	<10	365	<10	356	<10	374	<10	378	<10	378	<10
Foundation Year Doctor	520	51	526	15	565	<10	753	<10	582	<10	673	<10	633	31	634	29
Healthcare Support Worker	17,703	946	17,495	346	19,092	82	19,272	26	16,245	16	19,254	110	18,809	573	18,458	781
Hospital Nurse	17,836	1114	18,083	274	18,845	66	18,936	24	15,900	<10	18,832	64	18,488	414	18,173	603
Housekeeper	1518	47	1534	18	1541	<10	1491	<10	1470	<10	1462	<10	1431	28	1418	27
Laboratory Staff	2801	32	2826	<10	2809	<10	2898	<10	2564	<10	2930	10	2919	38	2884	47
Manager	3529	40	3502	<10	3530	<10	3490	<10	3165	<10	3562	<10	3536	30	3509	31
Medical Consultant	2591	72	2620	12	2648	<10	2578	<10	2293	<10	2682	10	2641	42	2628	41
Medical Secretary	1279	17	1294	<10	1289	<10	1290	<10	1287	<10	1287	<10	1281	<10	1265	21
Medical Student	292	<10	210	<10	170	<10	114	<10	61	<10	84	<10	84	<10	72	<10
Middle Grade Doctor	2777	111	2743	26	2849	<10	2881	<10	2565	<10	2979	11	2928	56	2853	83
Midwife	1481	22	1470	<10	1501	<10	1503	<10	1226	<10	1490	<10	1469	22	1459	19
Paramedic	1553	62	1594	10	1619	<10	1674	<10	1671	<10	1667	<10	1646	29	1639	42
Porter	1183	54	1142	<10	1233	<10	1228	<10	1010	<10	1197	<10	1170	26	1163	33
Student Hospital Nurse	1137	27	1144	19	1264	<10	972	<10	905	<10	872	<10	832	41	830	37
Sex																
Female	55,956	2281	55,726	648	58,545	174	58,812	49	50,008	23	58,712	231	57,792	1200	56,983	1595
Male	14,520	566	14,437	149	15,032	37	15,190	6	13,494	5	15,249	55	15,036	261	14,837	376
Age (years)^a^	43.4 ± 12.6	43.4 ± (11.8)	43.5 ± (12.5)	41.5 ± (12.4)	43.4 ± (12.6)	40.8 ± (12.5)	43.4 ± (12.6)	42.5 ± (12.1)	43.4 ± (12.6)	40.4 ± (15.7)	43.5 ± (12.6)	40.7 ± (12.7)	43.5 ± (12.6)	40.4 ± (12.2)	43.6 ± (12.6)	41 ± (12.2)
Welsh Index of Multiple Deprivation 2019 Quintiles																
1. Most deprived	10,255	540	10,284	145	10,930	40	11,007	<10	8846	<10	10,974	58	10,737	326	10,627	390
2	13,289	643	13,373	180	14,031	43	14,103	13	12,016	<10	14,049	73	13,743	359	13,571	438
3	14,096	514	13,884	163	14,614	43	14,730	10	12,597	<10	14,758	58	14,575	245	14,364	364
4	15,288	518	15,034	162	15,842	38	15,938	10	13,936	<10	15,908	49	15,736	241	15,479	362
5. Least deprived	17,548	632	17,588	147	18,160	47	18,224	13	16,107	<10	18,272	48	18,037	290	17,779	417

Individuals with multiple grouped staff roles are present in more than one category; the number of positive tests may therefore be overestimated for these individuals (approximately 4.5% of healthcare workers had multiple roles recorded). Cases where small counts within groups could be derived (<5) have been masked as <10. The employing organisations and entries where a count of <5 could not be masked have been omitted due to data governance requirements.^a^Values are given as mean ± SD.

### Logistic regression results

We calculated the odds ratios (ORs), with 95% confidence intervals (CIs), for univariable and multivariable logistic regression models for positive SARS-CoV-2 PCR tests, with the results displayed in Table 3. The univariable results indicated increased risk for staff who were patient-facing and for those in the following roles: Foundation year doctors, healthcare support workers, hospital nurses and student hospital nurses. The multivariable analyses showed similar results for the staff roles, with the exception of student hospital nurses, which suggests confounding for this group within the analyses. In general, individuals living in less-deprived areas were at a lower risk of infection.

**Table 3.** Logistic regression results for positive SARS-CoV-2 PCR tests.

Odds ratios (95% confidence interval)	Univariable	Multivariable patient-facing	Multivariable grouped staff roles
Age	**0.990 (0.988–0.991)**	**0.994 (0.992–0.996)**	**0.994 (0.993–0.996)**
Sex (reference female)			
Male	**0.901 (0.851–0.955)**	0.986 (0.929–1.046)	1.062 (0.996–1.133)
Patient-facing (reference non-patient-facing)			
Patient-facing	**2.474 (2.281–2.683)**	**2.278 (2.097–2.474)**	–
Undetermined	0.900 (0.784–1.032)	0.912 (0.794–1.048)	–
Grouped staff roles (dummy variables, reference no.)			
Allied Health Professionals	**0.601 (0.543–0.666)**	–	**0.618 (0.526–0.725)**
Call Handler	**0.633 (0.488–0.820)**	–	0.724 (0.513–1.023)
Clerical Worker	**0.459 (0.414–0.508)**	–	**0.502 (0.428–0.588)**
Community Nurse	**0.752 (0.654–0.864)**	–	**0.744 (0.627–0.884)**
Cook	**0.703 (0.508–0.972)**	–	0.771 (0.543–1.093)
Driver	**0.366 (0.217–0.618)**	–	**0.496 (0.284–0.865)**
Foundation Year Doctor	**2.124 (1.791–2.520)**	–	**1.825 (1.465–2.272)**
Healthcare Support Worker	**1.747 (1.668–1.831)**	–	**1.356 (1.196–1.538)**
Hospital Nurse	**1.475 (1.406–1.547)**	–	**1.270 (1.122–1.438)**
Housekeeper	**0.807 (0.677–0.961)**	–	**0.710 (0.573–0.880)**
Laboratory Staff	**0.451 (0.381–0.533)**	–	**0.478 (0.387–0.590)**
Manager	**0.305 (0.254–0.366)**	–	**0.363 (0.290–0.453)**
Medical Consultant	**0.647 (0.558–0.750)**	–	**0.667 (0.549–0.810)**
Medical Secretary	**0.380 (0.290–0.498)**	–	**0.388 (0.288–0.523)**
Medical Student	1.303 (0.827–2.052)	–	0.692 (0.433–1.105)
Middle Grade Doctor	0.976 (0.868–1.097)	–	0.911 (0.768–1.080)
Midwife	**0.444 (0.351–0.563)**	–	**0.430 (0.330–0.560)**
Paramedic	0.882 (0.751–1.034)	–	1.124 (0.797–1.585)
Porter	0.990 (0.828–1.184)	–	0.981 (0.786–1.223)
Student Hospital Nurse	**1.288 (1.086–1.527)**	–	**0.734 (0.615–0.877)**
Month of observation (Reference: April)			
May	**0.281 (0.260–0.304)**	**0.276 (0.255–0.299)**	**0.276 (0.255–0.299)**
June	**0.071 (0.062–0.082)**	**0.070 (0.061–0.080)**	**0.069 (0.060–0.080)**
July	**0.018 (0.014–0.024)**	**0.018 (0.014–0.023)**	**0.018 (0.014–0.023)**
August	**0.011 (0.008–0.016)**	**0.010 (0.007–0.015)**	**0.010 (0.007–0.015)**
September	**0.096 (0.085–0.108)**	**0.094 (0.083–0.106)**	**0.093 (0.082–0.105)**
October	**0.497 (0.466–0.529)**	**0.489 (0.459–0.521)**	**0.487 (0.456–0.519)**
November	**0.679 (0.641–0.720)**	**0.670 (0.632–0.711)**	**0.667 (0.629–0.708)**
Deprivation quintile (Reference 1: most deprived)			
2	**0.898 (0.838–0.963)**	**0.921 (0.859–0.989)**	0.950 (0.885–1.020)
3	**0.683 (0.635–0.735)**	**0.771 (0.715–0.831)**	**0.819 (0.759–0.883)**
4	**0.624 (0.579–0.671)**	**0.731 (0.678–0.789)**	**0.807 (0.747–0.871)**
5 (least deprived)	**0.624 (0.581–0.670)**	**0.677 (0.629–0.728)**	**0.787 (0.730–0.849)**
Organisation (dummy variables, reference no.)			
A	**0.769 (0.674–0.878)**	0.876 (0.733–1.046)	0.791 (0.582–1.077)
B	**0.257 (0.138–0.478)**	0.580 (0.306–1.097)	0.645 (0.338–1.230)
C	**0.252 (0.178–0.357)**	**0.456 (0.315–0.66)**	**0.522 (0.358–0.762)**
D	**0.586 (0.459–0.749)**	**0.733 (0.559–0.96)**	**0.753 (0.573–0.988)**
E	**2.075 (1.969–2.187)**	**1.810 (1.603–2.043)**	**1.772 (1.559–2.015)**
F	1.027 (0.962–1.097)	0.889 (0.781–1.012)	0.877 (0.766–1.005)
G	**1.326 (1.252–1.405)**	**1.279 (1.131–1.445)**	**1.259 (1.107–1.431)**
H	**0.486 (0.388–0.609)**	**0.541 (0.421–0.696)**	**0.542 (0.420–0.699)**
I	**0.836 (0.787–0.887)**	0.932 (0.815–1.066)	0.896 (0.779–1.030)
J	**0.885 (0.832–0.940)**	0.965 (0.850–1.096)	0.922 (0.807–1.054)
K	**0.569 (0.520–0.622)**	**0.604 (0.522–0.699)**	**0.597 (0.513–0.694)**
L	**2.390 (1.736–3.291)**	**2.318 (1.654–3.248)**	**1.689 (1.158–2.463)**
M	**0.360 (0.172–0.754)**	**0.448 (0.211–0.949)**	0.629 (0.295–1.345)
N	**0.384 (0.296–0.498)**	**0.511 (0.387–0.677)**	**0.611 (0.454–0.822)**
Intercept	–	0.033 (0.028–0.039)	0.060 (0.050–0.073)
Observations/individuals			
Observations	577,985	577,985	577,985
Individuals	77,587	77,587	77,587

Note: Results that were statistically significant at the 95% level are presented in bold font.

Foundation year doctors were at the highest risk of a positive SARS-CoV-2 test in both the univariable and multivariable analyses. We included a variable for the month of observation and found a statistically significant reduced risk for each month following the reference month, April. The randomised organisation variables also showed statistically significant ORs, indicating differences in risk of infection depending on organisation. The sensitivity analyses showed some variance for the observation month, which was consistent with the fixed effects models and did not impact the overall interpretation. There was no variance in random effects models with an individual level intercept term.

### Multilevel logistic regression sensitivity analyses

The multilevel models revealed very similar coefficient estimates to the logistic regression model. When including a random effect at the individual level there was an estimated variance of 0. The model with monthly observation included as a random effect indicated a statistically significant variance component. The results for the multilevel models with month and individual included as a random effect are displayed in Table S5 and Table S6, respectively.

## Discussion

Our national study of 77,587 healthcare workers found that patient-facing healthcare workers were at a higher risk of SARS-CoV-2 infection than non-patient-facing healthcare workers, with over 1 in every 25 patient-facing healthcare workers testing positive in April 2020 alone, and an overall adjusted OR of 2.28 (95% CI 2.10–2.47). The three staff groups at highest risk of testing positive were foundation year doctors, nursing staff and healthcare support workers. Factors contributing to this could include the frequent close-contact procedures carried out by these groups (e.g. performing throat swabs, clinical examinations and provision of personal care). These groups are also often ‘ward-based’, where there may be limited opportunity to socially distance due to crowded work and rest spaces.

Foundation year doctors (doctors one to two years post qualification) were the staff group at the highest risk of testing positive in our cohort, with an adjusted OR of 1.83 (95% CI 1.47–2.27). Possible explanations for this include increased movement between wards, closer proximity and extended contact with a greater number of patients at an earlier stage of their admission. During the first wave of the pandemic, foundation year doctors were frequently redeployed to unfamiliar acute medical wards, emergency departments and intensive care units from their otherwise more diverse placements. This increased their exposure to acutely unwell patients, who may have been infected with SARS-COV-2 but who may not have been tested or adequately segregated. The lack of testing and precise knowledge on the transmission of the virus during the first wave, coupled with variable PPE provision in the different parts of the secondary acute care hospital pathway could partly explain our findings. It is clear that the health service was not sufficiently prepared to protect staff at the beginning of the pandemic, though the relatively high positive test rate which has recurred with the autumn wave suggests ongoing susceptibility in healthcare workers.

We found a decreased risk of infection with increasing age in our study. This is consistent with a Danish study of SARS-CoV-2 seroprevalence in healthcare workers, which also found a lower infection rate in older healthcare workers.^22^ It is feasible that age interacts with the grouped roles and may be explained by older individuals taking roles with less patient contact, particularly among those with underlying health conditions, or adopting more risk averse behaviour in the workplace, for example stricter social distancing, hand hygiene and PPE use. As with other studies, decreasing deprivation was associated with decreased risk of infection in our study.^23–25^ This association with deprivation holds even after adjusting for staff role. It may be, therefore, that factors associated with deprivation that increase community transmission also impact healthcare workers.

Risk of SARS-CoV-2 infection changed over time, and varied between organisations, consistent with the changing community prevalence and varied geographic spread of SARS-CoV-2.^26,27^ This has important implications in that community prevalence may be associated with healthcare workers' risk of infection. Although there has been a policy of universal PPE use for all patient contact since mid-April, this has not prevented the resurgence of infection, and there is continued ambiguity as to whether wider access to higher grade PPE is required.^28^

### Strengths

We performed a large national study using eight months of data for over 77,000 healthcare workers in all NHS organisations across Wales. As well as patient-facing status, we also included staff roles to determine who was at the highest risk of testing positive with SARS-CoV-2. We also included a time-varying component to account for changes in the baseline risk of infection over time. Linked demographic information (age, sex, deprivation status) was also included to investigate factors associated with the risk of infection. Our study is the first to examine the effects of the viral resurgence in the UK on infection among healthcare workers and provides important data on the size of the problem in the second wave.

### Limitations

We were unable to account for the specific time at risk for individuals, as the data were limited to monthly updates of staff roles and organisations. We were also unable to determine which department individuals worked within, or if individuals had additional roles during the pandemic. Furthermore, many traditionally non-patient-facing roles may have changed to patient-facing to help ease pressures during the pandemic, or vice versa where alternative measures could be taken (e.g. virtual consultation). We did not investigate how the availability of testing has influenced the measured healthcare workers’ infection rates. Observed positivity rates could be significantly underestimated, as our data would not include asymptomatic infections unless healthcare workers were screened. Due to data limitations, we do not know what proportion of tests were ‘screening tests’ or tests taken by symptomatic individuals. Additionally, we did not account for people who are categorised in more than one staff group.

### Generalisability

Our results mirror previous studies on healthcare workers’ infection rates.^22,29^ In contrast to a similar large population-based study in Scotland, we have examined infection rates rather than hospitalisation.^13^ Although hospitalisation is an important patient-centred endpoint, given the ongoing constraints on HCW availability it is important to investigate and monitor HCW infection rates. Our results suggest that the increasing infection rates among healthcare workers (along with the necessary government policy for self-isolation to reduce further transmission) could significantly reduce the workforce, putting patient care in jeopardy. It is also notable that although our study cohort was relatively young, meaning that risk of hospitalisation or death due to infection should be low, long-term effects of COVID-19 infection are prevalent and could adversely affect the healthcare workforce in the longer term.^30^

### Conclusion and interpretation

We determined that patient-facing healthcare worker roles were at the highest risk of SARS-CoV-2 infection. We also found that after adjustment, foundation year doctors, healthcare support workers and hospital nurses were at the highest risk of infection among all staff groups. This has important policy implications for PPE provision and the prioritisation of vaccination. First, the provision of adequate PPE, regular refresher training and active enforcement of appropriate use among these frontline workers should be prioritised. Second, to maintain operational readiness and capability to respond to sudden increases in healthcare service demand, these healthcare workers should be prioritised in further vaccine rollouts.

## Supplemental Material

sj-pdf-1-jrs-10.1177_01410768221107119 - Supplemental material for SARS-CoV-2 infection risk among 77,587 healthcare workers: a national observational longitudinal cohort study in Wales, United Kingdom, April to November 2020 Supplemental material, sj-pdf-1-jrs-10.1177_01410768221107119 for SARS-CoV-2 infection risk among 77,587 healthcare workers: a national observational longitudinal cohort study in Wales, United Kingdom, April to November 2020 by Joe Hollinghurst, Laura North, Tamas Szakmany, Richard Pugh, Gwyneth A Davies, Shanya Sivakumaran, Rebecca Jarvis, Martin Rolles, W Owen Pickrell, Ashley Akbari, Gareth Davies, Rowena Griffiths, Jane Lyons, Fatemeh Torabi, Richard Fry, Mike B Gravenor and Ronan A Lyons in Journal of the Royal Society of Medicine

sj-pdf-2-jrs-10.1177_01410768221107119 - Supplemental material for SARS-CoV-2 infection risk among 77,587 healthcare workers: a national observational longitudinal cohort study in Wales, United Kingdom, April to November 2020 Supplemental material, sj-pdf-2-jrs-10.1177_01410768221107119 for SARS-CoV-2 infection risk among 77,587 healthcare workers: a national observational longitudinal cohort study in Wales, United Kingdom, April to November 2020 by Joe Hollinghurst, Laura North, Tamas Szakmany, Richard Pugh, Gwyneth A Davies, Shanya Sivakumaran, Rebecca Jarvis, Martin Rolles, W Owen Pickrell, Ashley Akbari, Gareth Davies, Rowena Griffiths, Jane Lyons, Fatemeh Torabi, Richard Fry, Mike B Gravenor and Ronan A Lyons in Journal of the Royal Society of Medicine

## Declarations

### Guarantor

JH.

### Provenance

Not commissioned; peer-reviewed by Julie Morris.
