# Supplementary material for: SARS-CoV-2 infection risk among 77,587 healthcare workers: a national observational longitudinal cohort study in Wales, United Kingdom, April to November 2020
Source: J R Soc Med. 2022 Jul 7;115(12):467–78. doi: 10.1177/01410768221107119 (PMC9747896; doi:10.1177/01410768221107119)
Supplement: sj-pdf-2-jrs-10.1177_01410768221107119 - Supplemental material for SARS-CoV-2 infection risk among 77,587 healthcare workers: a national observational longitudinal cohort study in Wales, United Kingdom, April to November 2020 [file sj-pdf-2-jrs-10.1177_01410768221107119.pdf]

SUPPLEMENTARY INFORMATION

Table S1. Breakdown of grouped roles

Grouped Role	Specific Role
Allied Health Professionals	Applied Psychologist - Clinical
	Applied Psychologist - Counselling
	Applied Psychologist - Forensic
	Applied Psychologist - Occupational
	Approved Mental Health Professional
	Assistant Psychologist
	Assistant Psychotherapist
	Child and Adolescent Psychological Therapist/Psychotherapist
	Chiropodist/Podiatrist
	Chiropodist/Podiatrist Manager
	Dietitian
	Dietitian Specialist Practitioner
	Multi Therapist
	Multi Therapist Consultant
	Occupational Therapist
	Occupational Therapy Specialist Practitioner
	Operating Department Practitioner
	Orthoptist
	Orthotist
	Physiotherapist
	Physiotherapist Consultant
	Physiotherapist Specialist Practitioner
	Prosthetist
	Prosthetist Specialist Practitioner
	Psychological Wellbeing Practitioner
	Psychotherapist
	Radiographer - Diagnostic
	Radiographer - Diagnostic, Consultant
	Radiographer - Diagnostic, Specialist Practitioner
	Radiographer - Therapeutic
	Radiographer - Therapeutic, Consultant
	Radiographer - Therapeutic, Specialist Practitioner
	Speech and Language Therapist
	Speech and Language Therapist Consultant
	Speech and Language Therapist Specialist Practitioner
Call Handler	Call Operator
	Emergency Medical Dispatcher
	Non-Emergency Call Handler
	Non-Emergency Medical Dispatcher
	Telephonist
Clerical Worker	Clerical Worker
Community Nurse	Community Nurse

Cook	Cook
Driver	Driver
Foundation Year Doctor	Foundation Year 1<br>Foundation Year 2<br>Trust Grade Doctor - Foundation Level
Healthcare Support Worker	Health Care Support Worker<br>Healthcare Assistant
Hospital Nurse	Enrolled Nurse<br>Sister/Charge Nurse<br>Specialist Nurse Practitioner<br>Staff Nurse
Housekeeper	Cleaner<br>Housekeeper
Laboratory Staff	Consultant Healthcare Scientist<br>Healthcare Science Assistant<br>Healthcare Science Associate<br>Healthcare Science Practitioner<br>Healthcare Scientist<br>Specialist Healthcare Science Practitioner<br>Specialist Healthcare Scientist
Manager	Manager<br>Senior Manager
Medical Consultant	Consultant
Medical Secretary	Medical Secretary
Medical Student	Medical Student
Middle Grade Doctor	Associate Specialist (Closed to new entrants)<br>Senior House Officer<br>Specialist Registrar<br>Specialty Doctor<br>Specialty Registrar<br>Trust Grade Doctor - Career Grade level<br>Trust Grade Doctor - Specialty Registrar
Midwife	Midwife<br>Midwife - Consultant
Paramedic	Ambulance Care Assistant/Patient Transport Service Driver<br>Paramedic<br>Paramedic Specialist Practitioner
Porter	Porter
Student Hospital Nurse	Student Nurse - Adult Branch<br>Student Nurse - Child Branch

Table S2. Patient-facing groups by grouped role

**Grouped Role**	**Patient-facing**	**Non Patient-facing**	**Undetermined**
Allied Health Professionals			Yes
Call Handler		Yes	
Clerical Worker		Yes	
Community Nurse	Yes		

Cook		Yes	
Driver	Yes		
Foundation Year Doctor	Yes		
Healthcare Support Worker	Yes		
Hospital Nurse	Yes		
Housekeeper			Yes
Laboratory Staff		Yes	
Manager			Yes
Medical Consultant	Yes		
Medical Secretary		Yes	
Medical Student	Yes		
Middle Grade Doctor	Yes		
Midwife	Yes		
Paramedic	Yes		
Porter	Yes		
Student Hospital Nurse	Yes		

Table S3a. Number of individuals per month for those with and without demographic information

Month	Positive COVID test	Complete demographics	All data
Apr	No	70476	77402
Apr	Yes	2847	3083
May	No	70163	76873
May	Yes	797	890
Jun	No	73577	80817
Jun	Yes	211	225
Jul	No	74002	81310
Jul	Yes	55	62
Aug	No	Removed due to governance restrictions	
Aug	Yes		
Sep	No	73961	81329
Sep	Yes	286	312
Oct	No	72828	80105
Oct	Yes	1461	1574
Nov	No	71820	78999
Nov	Yes	1971	2141

Table S3b. Chi-squared tests for the differences in proportions between tests in those with and without demographic information

Month	*Chi-squared statistic, degrees of freedom (df), and p-value.*
April	X-squared = 0.26911, df = 1, p-value = 0.6039
May	X-squared = 0.13217, df = 1, p-value = 0.7162
June	X-squared = 0.067896, df = 1, p-value = 0.7944

July	X-squared = 0.0020886, df = 1, p-value = 0.9635
August	X-squared = 0.022408, df = 1, p-value = 0.881
September	X-squared = 0.0031304, df = 1, p-value = 0.9554
October	X-squared = 0.29914, df = 1, p-value = 0.5844
November	X-squared = 0.14481, df = 1, p-value = 0.7035

Table S4. Longitudinal positive SARS-CoV-2 test rates amongst NHS employed healthcare workers in Wales (UK). The percentages were calculated for each category using the number of people testing positive as a proportion of healthcare workers in each category. Where the number of individuals in the group were less than 10 a less than sign (<) has been used to mask exact values for data governance requirements.

**Month of Observation**	Apr	May	Jun	Jul	Aug	Sep	Oct	Nov
**Total Healthcare Workers (N)**	73,323	70,960	73,788	74,057	63,530	74,247	74,289	73,791
**Healthcare workers testing positive**	2,847	797	211	55	28	286	1,461	1,971
**SARS-CoV-2 PCR Positive Rates**								
Percentage of Healthcare workers testing positive	3.88%	1.12%	0.29%	0.07%	0.04%	0.39%	1.97%	2.67%
**Patient-facing**								
Patient-facing	4.73%	1.41%	0.34%	0.09%	-	0.41%	2.23%	3.15%
Non-Patient-facing	1.41%	0.28%	0.12%	<0.08%	-	0.38%	1.33%	1.45%
Undetermined	1.65%	0.44%	0.15%	<0.15%	-	0.23%	1.00%	1.08%
**Grouped Staff Roles**								
Allied Health Professionals	2.44%	0.84%	0.32%	<0.17%	<0.20%	0.16%	0.99%	1.69%
Call Handler	3.73%	<1.15%	<1.16%	<1.16%	<1.16%	<1.18%	1.51%	<1.13%
Clerical Worker	1.24%	0.31%	<0.13%	<0.13%	<0.16%	0.40%	1.46%	1.48%
Community Nurse	3.06%	0.63%	<0.38%	<0.38%	<0.48%	<0.38%	1.26%	2.38%
Cook	3.59%	<1.99%	<1.95%	<2.00%	<2.07%	<2.02%	<1.95%	<2.01%
Driver	<2.81%	<3.33%	<2.75%	<2.67%	<2.73%	<2.60%	<2.58%	<2.58%
Foundation Year Doctor	8.93%	2.77%	<1.74%	<1.31%	<1.69%	<1.46%	4.67%	4.37%
Healthcare Support Worker	5.07%	1.94%	0.43%	0.13%	0.10%	0.57%	2.96%	4.06%
Hospital Nurse	5.88%	1.49%	0.35%	0.13%	<0.06%	0.34%	2.19%	3.21%
Housekeeper	3.00%	1.16%	<0.64%	<0.67%	<0.68%	<0.68%	1.92%	1.87%
Laboratory Staff	1.13%	<0.35%	<0.35%	<0.34%	<0.39%	0.34%	1.29%	1.60%
Manager	1.12%	<0.28%	<0.28%	<0.29%	<0.31%	<0.28%	0.84%	0.88%
Medical Consultant	2.70%	0.46%	<0.38%	<0.39%	<0.43%	0.37%	1.57%	1.54%
Medical Secretary	1.31%	<0.77%	<0.77%	<0.77%	<0.77%	<0.77%	<0.77%	1.63%
Medical Student	<3.31%	<4.55%	<5.56%	<8.06%	<14.08%	<10.64%	<10.64%	<12.20%
Middle Grade Doctor	3.84%	0.94%	<0.35%	<0.35%	<0.39%	0.37%	1.88%	2.83%
Midwife	1.46%	<0.68%	<0.66%	<0.66%	<0.81%	<0.67%	1.48%	1.29%
Paramedic	3.84%	0.62%	<0.61%	<0.59%	<0.59%	<0.60%	1.73%	2.50%
Porter	4.37%	<0.87%	<0.80%	<0.81%	<0.98%	<0.83%	2.17%	2.76%
Student Hospital Nurse	2.32%	1.63%	<0.78%	<1.02%	<1.09%	<1.13%	4.70%	4.27%
**Sex**								
Female	3.92%	1.15%	0.30%	0.08%	0.05%	0.39%	2.03%	2.72%
Male	3.75%	1.02%	0.25%	0.04%	0.04%	0.36%	1.71%	2.47%
**Welsh Index of Multiple Deprivation 2019**								
1. Most Deprived	5.00%	1.39%	0.36%	<0.09%	<0.11%	0.53%	2.95%	3.54%
2	4.62%	1.33%	0.31%	0.09%	<0.08%	0.52%	2.55%	3.13%
3	3.52%	1.16%	0.29%	0.07%	<0.08%	0.39%	1.65%	2.47%
4	3.28%	1.07%	0.24%	0.06%	<0.07%	0.31%	1.51%	2.29%
5. Least Deprived	3.48%	0.83%	0.26%	0.07%	<0.06%	0.26%	1.58%	2.29%

Multilevel logistic regression models: Observation month included as a random effect

The multilevel model with the monthly observation included as a random effect indicated a statistically significant variance component. This indicated that including the observation month was an important consideration for the logistic regression model presented in the main results (Table 3.).

Table S5. Multilevel logistic regression model. Observation month included as a random effect.

Odds Ratios (95% Confidence Interval)	Univariable	Patient-facing	Grouped Staff Roles
**Age**			
Age	0.994 (0.992,0.996)	0.994 (0.992,0.996)	0.995 (0.993,0.996)
**Gender (Reference: Female)**			
Gender:Male	0.986 (0.93,1.047)	0.986 (0.93,1.047)	1.061 (0.995,1.132)
**Patient Facing (Reference: Non-patient-facing)**			
Patient -facing	2.255 (2.076,2.45)	2.255 (2.076,2.45)	-
Undetermined	0.913 (0.795,1.049)	0.913 (0.795,1.049)	-
**Grouped Staff Roles**			
Allied Health Professionals	0.601 (0.542,0.666)	-	0.621 (0.529,0.728)
Call Handler	0.637 (0.491,0.827)	-	0.727 (0.516,1.024)
Clerical Worker	0.457 (0.412,0.507)	-	0.505 (0.431,0.592)
Community Nurse	0.745 (0.647,0.857)	-	0.747 (0.628,0.888)
Cook	0.706 (0.51,0.979)	-	0.772 (0.544,1.096)
Driver	0.37 (0.218,0.626)	-	0.498 (0.286,0.868)
Foundation Year Doctor	2.208 (1.865,2.614)	-	1.798 (1.444,2.238)
Healthcare Support Worker	1.755 (1.674,1.839)	-	1.347 (1.188,1.528)
Hospital Nurse	1.467 (1.398,1.54)	-	1.264 (1.117,1.43)
Housekeeper	0.815 (0.684,0.971)	-	0.714 (0.576,0.884)
Laboratory Staff	0.452 (0.382,0.535)	-	0.482 (0.39,0.594)
Manager	0.305 (0.254,0.366)	-	0.366 (0.292,0.458)
Medical Consultant	0.643 (0.554,0.747)	-	0.67 (0.551,0.814)
Medical Secretary	0.386 (0.294,0.506)	-	0.392 (0.291,0.527)
Medical Student	1.02 (0.609,1.707)	-	0.697 (0.418,1.162)
Middle Grade Doctor	0.975 (0.866,1.097)	-	0.912 (0.769,1.081)
Midwife	0.442 (0.348,0.561)	-	0.433 (0.332,0.566)
Paramedic	0.9 (0.767,1.056)	-	1.122 (0.797,1.579)
Porter	0.984 (0.822,1.179)	-	0.98 (0.786,1.223)
Student Hospital Nurse	1.294 (1.09,1.536)	-	0.739 (0.619,0.881)
**Organisation**			
A	0.877 (0.734,1.047)	0.877 (0.734,1.047)	0.793 (0.583,1.078)
B	0.58 (0.306,1.098)	0.58 (0.306,1.098)	0.646 (0.338,1.232)
C	0.458 (0.316,0.662)	0.458 (0.316,0.662)	0.524 (0.359,0.763)
D	0.735 (0.562,0.962)	0.735 (0.562,0.962)	0.755 (0.575,0.991)
E	1.787 (1.583,2.017)	1.787 (1.583,2.017)	1.749 (1.539,1.987)
F	0.89 (0.78,1.015)	0.89 (0.78,1.015)	0.879 (0.765,1.008)
G	1.273 (1.126,1.438)	1.273 (1.126,1.438)	1.253 (1.102,1.424)
H	0.545 (0.424,0.701)	0.545 (0.424,0.701)	0.546 (0.423,0.704)

I	0.933 (0.816,1.066)	0.933 (0.816,1.066)	0.897 (0.78,1.031)
J	0.965 (0.85,1.096)	0.965 (0.85,1.096)	0.923 (0.808,1.054)
K	0.608 (0.525,0.703)	0.608 (0.525,0.703)	0.6 (0.516,0.698)
L	2.28 (1.654,3.144)	2.28 (1.654,3.144)	1.668 (1.161,2.396)
M	0.449 (0.203,0.996)	0.449 (0.203,0.996)	0.629 (0.282,1.404)
N	0.514 (0.389,0.681)	0.514 (0.389,0.681)	0.613 (0.456,0.825)
**WIMD (Reference 1. Most Deprived)**			
2	0.923 (0.861,0.99)	0.923 (0.861,0.99)	0.951 (0.887,1.021)
3	0.775 (0.719,0.835)	0.775 (0.719,0.835)	0.822 (0.762,0.886)
4	0.735 (0.682,0.793)	0.735 (0.682,0.793)	0.81 (0.75,0.874)
5.Least Deprived	0.681 (0.634,0.732)	0.681 (0.634,0.732)	0.791 (0.734,0.853)
Intercept	0.011 (0.005,0.022)	0.011 (0.005,0.022)	0.019 (0.009,0.04)
Random effects			
Constant	-	1 (1,1)	1 (1,1)
Variance	-	1.069 (0.022,2.115)	1.072 (0.022,2.121)
-	-	-	-
Observations	577,985	577,985	577,985
Groups (Months)	8	8	8

Multilevel logistic regression models: Individual included as a random effect

The multilevel model with the individual included as a random effect indicated a variance component of 0.

Table S6. Multilevel logistic regression model with a random intercept at the individual level.

**Odds Ratios (95% Confidence Interval)**	**Multivariable Patient-facing**	**Multivariable Grouped Staff Roles**
Age	0.994 (0.992,0.996)	0.995 (0.993,0.997)
Sex (Reference Female)		
Male	0.987 (0.93,1.047)	1.062 (0.996,1.132)
**Patient-facing (Reference Non-Patient-facing)**		
Patient-facing	2.251 (2.074,2.444)	-
Undetermined	0.916 (0.798,1.051)	-
**Grouped Staff Roles (Dummy Variables, Reference No)**		
Allied Health Professionals	-	0.625 (0.534,0.732)
Call Handler	-	0.733 (0.521,1.032)
Clerical Worker	-	0.508 (0.434,0.594)
Community Nurse	-	0.752 (0.634,0.892)
Cook	-	0.785 (0.554,1.111)
Driver	-	0.499 (0.287,0.869)
Foundation Year Doctor	-	1.771 (1.423,2.206)
Healthcare Support Worker	-	1.346 (1.188,1.524)
Hospital Nurse	-	1.276 (1.13,1.442)
Housekeeper	-	0.726 (0.587,0.898)
Laboratory Staff	-	0.485 (0.394,0.598)
Manager	-	0.369 (0.295,0.461)
Medical Consultant	-	0.672 (0.554,0.815)
Medical Secretary	-	0.397 (0.295,0.534)
Medical Student	-	0.874 (0.549,1.39)
Middle Grade Doctor	-	0.924 (0.78,1.094)
Midwife	-	0.437 (0.336,0.569)
Paramedic	-	1.11 (0.79,1.56)
Porter	-	0.99 (0.796,1.232)
Student Hospital Nurse	-	0.758 (0.636,0.904)
**Organisation (Dummy Variables, Reference No)**		
A	0.885 (0.742,1.056)	0.808 (0.596,1.096)
B	0.588 (0.311,1.11)	0.654 (0.343,1.245)
C	0.464 (0.321,0.671)	0.53 (0.364,0.771)
D	0.745 (0.57,0.973)	0.765 (0.583,1.002)
E	1.781 (1.581,2.007)	1.737 (1.532,1.971)
F	1.033 (0.909,1.174)	1.017 (0.889,1.163)
G	1.284 (1.138,1.448)	1.259 (1.109,1.429)
H	0.551 (0.429,0.708)	0.552 (0.429,0.711)
I	0.939 (0.823,1.071)	0.901 (0.785,1.034)

J	0.973 (0.858,1.102)	0.928 (0.814,1.058)
K	0.613 (0.531,0.709)	0.605 (0.521,0.703)
L	2.07 (1.483,2.89)	1.446 (0.99,2.112)
M	0.51 (0.241,1.081)	0.714 (0.335,1.523)
N	0.52 (0.393,0.687)	0.617 (0.459,0.828)
**Deprivation Quintile (Reference 1. Most Deprived)**		
2	0.925 (0.863,0.992)	0.953 (0.888,1.022)
3	0.777 (0.721,0.837)	0.822 (0.763,0.886)
4	0.737 (0.684,0.794)	0.81 (0.751,0.874)
5. Least Deprived	0.683 (0.636,0.734)	0.791 (0.734,0.852)
**Intercept**	0.01 (0.009,0.012)	0.019 (0.016,0.023)
**Random Effects**		
Constant	1 (1,1)	1 (1,1)
Variance	0 (0,0)	0 (0,0)
-	-	-
Observations	577985	577985
Individuals	77587	77587
